# Impact of ABO blood group on NEC incidence and mortality in VLBW infants

**DOI:** 10.1038/s41390-025-04181-z

**Published:** 2025-06-07

**Authors:** Wenhan Yue, Yuhan Liu, Haifeng Zong, Xuemei Huang, Yongyan Shi

**Affiliations:** 1 Division of Neonatology, Department of Pediatrics, Shengjing Hospital of China Medical University, Shenyang, Liaoning China; 2 Department of Neonatology, Shenzhen Maternity and Child Healthcare Hospital, Shenzhen, China; 3 Department of Neonatology, Liuzhou Maternity & Child Healthcare Hospital, Liuzhou, Guangxi China

## Abstract

**Background:**

This study aimed to investigate the relationship between ABO blood group and the incidence of necrotizing enterocolitis (NEC) or mortality in very low birth weight (VLBW) infants.

**Methods:**

A retrospective single-center cohort study was conducted on VLBW infants admitted to Shengjing Hospital of China Medical University from 2014 to 2023. Bell’s staging system was used to define NEC severity, and deaths were recorded. Confirmed NEC with Bell’s stage ≥2 and mortality were defined as primary outcomes and compared among the four ABO blood groups.

**Results:**

The primary composite outcome occurred in 14.7% (847/5774). Among the 5774 VLBW infants enrolled, the overall mortality was 11.0% (635/5774). Confirmed NEC (Bell’s stage ≥2) occurred in 5.0% (288/5774) of the cohort, with NEC-related mortality of 21.2% (61/288). ABO blood groups were not associated with mortality and the incidence of NEC in VLBW infants. These findings remained consistent after adjusting for perinatal factors, maternal factors, and admission year.

**Conclusion:**

This study suggests no significant association between ABO blood group and NEC incidence, severity, or mortality in VLBW infants, contrasting with previous reports. Variations in study populations, definition of primary outcomes, statistical methods, and transfusion strategies may explain the differing findings.

**Impact:**

- ABO blood groups have no influence on the incidence of confirmed NEC or mortality in VLBW infants.
- Variations in study populations, definitions of primary outcomes, and statistical methods may explain discrepancies between our findings and previous reports.
- Transfusion strategies may also confound the relationship between ABO blood group and NEC.

## Introduction

Necrotizing enterocolitis (NEC) is a life-threatening gastrointestinal disorder primarily affecting very low birth weight (VLBW) infants. It is characterized by inflammation and necrosis of the small intestine and colon,^1^ often resulting in sepsis, peritonitis, perforation, and death.^2,3^ Despite intensive conservative management, ~40–50% of NEC cases require surgical intervention, with a mortality rate of around 25%.^4^ Factors, such as preterm birth, low birth weight, formula feeding, dysbiosis, and intestinal ischemia, contribute to the pathogenesis of NEC; however, the precise mechanisms remain unclear.^5,6^

In 2012, Thomson et al. reported an association between blood group AB and increased NEC mortality.^7^ Since then, several studies suggested differential susceptibility to gastrointestinal diseases among ABO blood groups.^8–11^ These studies suggested that ABO blood group antigens, which are expressed not only on red blood cells (RBCs) but also on the surfaces of the small and large intestines, might predispose certain blood groups to gut inflammation and NEC.

In contrast, our recent clinical observations yielded results that differ significantly from these earlier findings. This study aims to present our findings on the incidence of NEC and its associated complications, and mortality across ABO blood groups and propose an explanation for the observed discrepancies. Drawing on data from a national-level regional perinatal care center, our results may contribute to a deeper understanding of the relationship between ABO blood groups and NEC and provide a foundation for future investigations.

## Methods

### Study population

This retrospective cohort study included VLBW (<1500 g at birth) infants admitted to Shengjing Hospital of China Medical University between January 1, 2014, and December 31, 2023. Cases with major congenital anomalies, metabolic disorders, outborn status, and deaths or transfers within 24 h postnatally were excluded. Data were collected on sex, gestational age (GA), birth weight (BW), Apgar scores, maternal age, and perinatal complications and were grouped by ABO blood group.

### Definitions

BW *z*-score was calculated to quantify the deviation of an infant’s BW from the mean BW specific to GA and sex, based on the 2013 Fenton growth curve.^12^ Small for gestational age (SGA): defined as a BW less than the 10th percentile for gestational age according to Chinese neonatal BW values.^13^ Antenatal steroids: defined as a partial or complete course of antenatal steroids administered during the perinatal period. Chorioamnionitis: identified through clinical examination or histopathological assessment. NEC: diagnosed based on Bell’s staging of clinical and radiological findings.^14^ Surgical intervention for NEC was indicated for clinical deterioration, intestinal perforation, or stenosis following medical management.^15^ NEC-related death was defined as mortality directly attributable to NEC or associated with decisions for palliative care. Bronchopulmonary dysplasia (BPD): diagnosed according to the consensus established at the 2018 workshop.^16^ This study mainly considers moderate or severe BPD (≥grade 2). Intraventricular hemorrhage (IVH): defined and graded according to Papile criteria on cranial ultrasound or magnetic resonance imaging (MRI).^17^ This study mainly considers severe IVH (≥grade 3). Periventricular leukomalacia (PVL): defined by the detection of periventricular cysts on cranial ultrasound or MRI.^18^ This study mainly considers cystic periventricular leukomalacia (cPVL). Retinopathy of prematurity (ROP): defined and staged according to the International Classification of ROP.^19^ This study mainly considers severe ROP (≥grade 3). The incidence of above-mentioned complications was summarized and analyzed by ABO blood group.

### Outcomes

The primary outcome was a composite of NEC ≥ stage 2 or death. The incidence of NEC ≥ stage 2 and mortality were also analyzed, respectively. In subgroup analysis, the percentage of severe NEC (≥ stage 3), surgical NEC, and mortality was investigated in different blood groups.

### Statistical analysis

Baseline characteristics were summarized as means with standard deviations or medians with interquartile ranges (IQR) for continuous variables, depending on data distribution, and as counts with percentages for categorical variables. These characteristics were presented across blood type groups without inter-group comparisons.

To investigate the association between ABO blood type and outcomes (NEC or death, NEC alone, and death alone), univariable logistic regression and three multivariable-adjusted logistic regression models with stepwise adjustments were used. Model 1: adjusted for perinatal factors (sex, GA, BW *z*-score, multiple gestation, antenatal corticosteroid use, premature rupture of membranes, chorioamnionitis, and asphyxia) based on clinical relevance. Model 2: added maternal factors (maternal age, gestational diabetes mellitus, and pregnancy-induced hypertension). Model 3: further adjusted for admission year to account for advancements in clinical practices over time.

Subgroup analyses were pre-specified based on GA (<28 weeks or ≥28 weeks) and BW (<1000 g or 1000–1499 g). Sensitivity analyses were performed after excluding infants who died or were discharged against medical advice within 7 and 14 days of life, respectively.

To explore the impact of ABO blood type on confirmed NEC cases, we calculated the proportions of stage 3 NEC, surgical NEC, and mortality. Odds ratios (ORs) and 95% confidential internal (CI) for these outcomes were calculated using blood type O as the reference, with adjustments based on Model 3. Additionally, to align with prior studies comparing a specific blood type against the other three combined, similar analyses were conducted, reporting the incidence of primary outcomes (NEC or death) and adjusted odds ratios (aORs). All statistical analyses were performed using Stata software (StataCorp, TX).

## Results

### General data

**Fig. 1:**
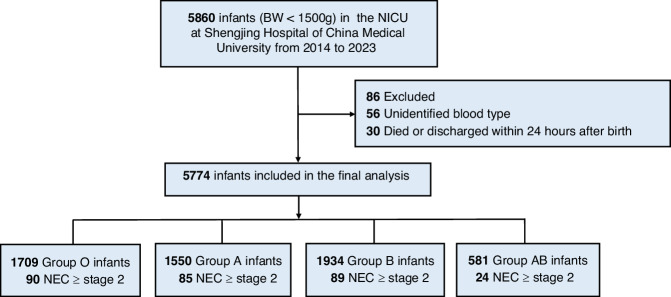
Flowchart of the study. NICU neonatal intensive care unit, NEC necrotizing enterocolitis.

Between January 1, 2014, and December 31, 2023, a total of 5860 VLBW infants were admitted, of which 5774 were ultimately included in the study. The patient enrollment flow was illustrated in Fig. 1. The median GA was 30 weeks (IQR: 28, 31), and the median BW was 1230 g (IQR: 1050, 1370). Based on Fenton’s reference values, the *z*-score for BW by GA was −0.59 (IQR: −1.21, 0.03).^12^ Male infants constituted 51.1% of the cohort. Cesarean section accounted for 73.3% of births, 26.8% of infants were classified as SGA, and 30.0% were from multiple births. The blood group distribution was as follows: group O (29.6%), group A (26.8%), group B (33.5%), and group AB (10.1%) (Table 1).

**Table 1:** Basic clinical characteristics of all VLBW participants with different blood groups.

Variables	Blood group O	Blood group A	Blood group B	Blood group AB	Total
	(*n* = 1709)	(*n* = 1550)	(*n* = 1934)	(*n* = 581)	(*n* = 5774)
Male	877 (51.3%)	794 (51.2%)	984 (50.9%)	296 (50.9%)	2951 (51.1%)
GA, median (IQR)	30 (28, 31)	30 (28, 31)	30 (28, 31)	30 (28, 31)	30 (28, 31)
GA < 28 weeks	214 (12.5%)	252 (16.3%)	229 (11.8%)	69 (11.9%)	764 (13.2%)
GA ≥ 28 weeks	1495 (87.5%)	1298 (83.7%)	1705 (88.2%)	512 (88.1%)	5010 (86.8%)
BW, median (IQR)	1230 (1053, 1370)	1219 (1030, 1360)	1240 (1063, 1380)	1235 (1040, 1370)	1230 (1050, 1370)
BW < 1000 g	345 (20.2%)	341 (22.0%)	358 (18.5%)	124 (21.3%)	1168 (20.2%)
BW 1000–1499 g	1364 (79.8%)	1209 (78.0%)	1576 (81.5%)	457 (78.7%)	4606 (79.8%)
BW *z*-score, median (IQR)	−0.57 (−1.24, 0.01)	−0.54 (−1.18, 0.07)	−0.64 (−1.22, 0.01)	−0.62 (−1.26, −0.02)	−0.59 (−1.21, 0.03)
SGA	471 (27.6%)	395 (25.5%)	522 (27.0%)	161 (27.7%)	1549 (26.8%)
Apgar score <7 at 5 min	31 (1.8%)	37 (2.4%)	48 (2.5%)	13 (2.2%)	129 (2.2%)
Maternal age, median (IQR)	31 (28, 35)	31 (28, 35)	31 (28, 35)	31 (28, 35)	31 (28, 35)
Cesarean section	1243 (72.8%)	1111 (71.7%)	1446 (74.8%)	429 (73.8%)	4229 (73.3%)
Multiple gestation	523 (30.6%)	443 (28.6%)	565 (29.2%)	199 (34.3%)	1730 (30.0%)
Antenatal steroids	636 (37.2%)	509 (32.8%)	656 (33.9%)	225 (38.7%)	2026 (35.1%)
Gestational diabetes mellitus	296 (17.3%)	247 (15.9%)	325 (16.8%)	111 (19.1%)	979 (17.0%)
Gestational hypertension	675 (39.5%)	625 (40.3%)	783 (40.5%)	249 (42.9%)	2332 (40.4%)
PROM > 24 h	286 (16.7%)	253 (16.3%)	310 (16.0%)	95 (16.4%)	944 (16.4)
Chorioamnionitis	141 (8.3%)	147 (9.5%)	172 (8.9%)	42 (7.2%)	502 (8.7%)
NEC ≥ stage 2	90 (5.3%)	85 (5.5%)	89 (4.6%)	24 (4.1%)	288 (5.0%)
Surgical NEC	56 (3.3%)	48 (3.1%)	57 (2.9%)	15 (2.6%)	176 (3.1%)
NEC death	18 (1.1%)	23 (1.5%)	17 (0.9%)	3 (0.5%)	61 (1.1%)
msBPD^a^	205 (14.8%)	178 (14.2%)	219 (14.4%)	72 (15.4%)	674 (14.6%)
sIVH	24 (1.4%)	32 (2.1%)	23 (1.2%)	8 (1.4%)	87 (1.5%)
cPVL	62 (3.6%)	68 (4.4%)	80 (4.1%)	24 (4.1%)	234 (4.1%)
sROP^b^	41 (2.6%)	51 (3.6%)	44 (2.5%)	8 (1.5%)	144 (2.7%)
Mortality	180 (10.5%)	174 (11.2%)	208 (10.8%)	73 (12.6%)	635 (11.0%)
NEC ≥ stage 2 or death	248 (14.5%)	232 (15.0%)	274 (14.2%)	93 (16.0%)	847 (14.7%)
Death or major morbidities	476 (27.9%)	451 (29.1%)	529 (27.4%)	176 (30.3%)	1632 (28.3%)

*GA* gestational age, *IQR* interquartile range, *BW* birth weight, *SGA* small for gestational age, *PROM* premature rupture of the membrane, *NEC* necrotizing enterocolitis, *msBPD* moderate or severe bronchopulmonary dysplasia (≥grade 2), *sIVH* severe intraventricular hemorrhage (≥grade 3), *cPVL* cystic periventricular leukomalacia, *sROP* retinopathy of prematurity (≥grade 3).

^a^All BPD cases were retrospectively rediagnosed based on the medical records according to the BPD criteria proposed by NICHD Workshop in 2018, the diagnosis is only for gestational age <32 weeks.

^b^5269 cases of VLBW infants completed ROP screening.

### Association of ABO blood group with primary composite outcome

The incidence of the general outcomes is summarized in Table 1. The primary outcome, defined as death or incidence of confirmed NEC, occurred in 14.7% of the population (847/5774). The overall mortality of the cohort was 11.0% (635/5774). Confirmed NEC (Bell’s stage ≥2) occurred in 5.0% (288/5774) of the cohort, with NEC-related mortality of 21.2% (61/288). Infants with blood group AB exhibited a relatively lower incidence of NEC but higher mortality and a higher rate of the primary composite outcome compared to other blood groups, as shown in Table 1.

After adjusting for infant demographics and perinatal characteristics (Model 1), no significant differences were observed among blood groups A, B, or AB compared to blood group O. aOR were 1.00 (95% CI: 0.81, 1.22) for group A, 0.99 (95% CI: 0.81, 1.20) for group B, and 1.15 (95% CI: 0.88, 1.50) for group AB. Results remained consistent after further adjustments for maternal factors (Model 2) and admission year (Model 3), demonstrating the robustness of the findings, as shown in Table 2.

**Table 2:** Associations of outcomes with blood group in VLBW infants.

Outcomes	Univariable model	Multivariable model 1	Multivariable model 2	Multivariable model 3
	aOR (95% CI)	aOR (95% CI)	aOR (95% CI)	aOR (95% CI)
NEC ≥ stage 2 or death				
Blood group O	Reference	Reference	Reference	Reference
Blood group A	1.03 (0.85, 1.26)	1.00 (0.81, 1.22)	1.00 (0.81, 1.22)	1.00 (0.81, 1.22)
Blood group B	0.97 (0.81, 1.17)	0.99 (0.81, 1.20)	0.98 (0.81, 1.19)	0.98 (0.81, 1.19)
Blood group AB	1.12 (0.87, 1.46)	1.15 (0.88, 1.50)	1.16 (0.88, 1.51)	1.16 (0.89, 1.52)
NEC ≥ stage 2				
Blood group O	Reference	Reference	Reference	Reference
Blood group A	1.04 (0.77, 1.42)	1.01 (0.74, 1.37)	1.01 (0.74, 1.37)	1.00 (0.74, 1.36)
Blood group B	0.87 (0.64, 1.17)	0.88 (0.65, 1.19)	0.88 (0.65, 1.19)	0.88 (0.65, 1.19)
Blood group AB	0.78 (0.49, 1.23)	0.78 (0.49, 1.24)	0.78 (0.49, 1.24)	0.76 (0.48, 1.21)
Death				
Blood group O	Reference	Reference	Reference	Reference
Blood group A	1.07 (0.86, 1.34)	1.03 (0.82, 1.30)	1.03 (0.82, 1.31)	1.04 (0.82, 1.31)
Blood group B	1.02 (0.83, 1.26)	1.04 (0.84, 1.30)	1.04 (0.83, 1.30)	1.04 (0.83, 1.30)
Blood group AB	1.22 (0.91, 1.63)	1.26 (0.93, 1.70)	1.27 (0.94, 1.72)	1.30 (0.96, 1.77)

Multivariable model 1 included infant and perinatal characteristics of sex, gestational age, birth weight *z*-score, multiple gestation, antenatal corticosteroid, premature rupture of the membrane, chorioamnionitis, asphyxia.

Multivariable model 2 included model 1 plus maternal characteristics of maternal age, gestational diabetes mellitus, and gestational hypertension.

Multivariable model 3 included model 2 plus admission year.

*NEC* necrotizing enterocolitis, *aOR* adjusted odds ratio, *CI* confidential internal.

**Fig. 2:**
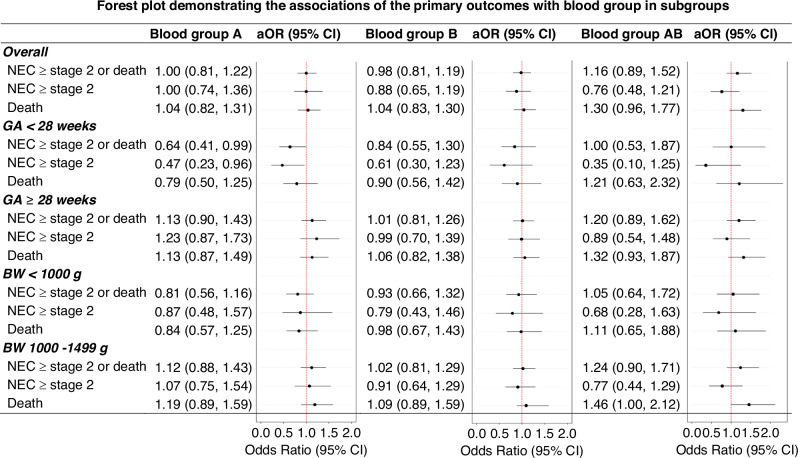
Forest plot demonstrating the associations of the primary outcomes with blood group in subgroups. The reference group for the plot is blood group O. Higher odds ratios indicate an increased likelihood of the outcomes. The adjusted odds ratios (aOR) and their 95% confidence intervals (CI) account for all infant characteristics, perinatal and maternal factors, as well as the year of admission. GA gestational age, BW birth weight, NEC necrotizing enterocolitis, aOR adjusted odds ratio.

Logistic regression comparing each blood group with the other three combined also revealed no significant differences in primary outcomes across models, as shown in Table 3. Subgroup analysis showed that the aOR for confirmed NEC or mortality in infants with GA < 28 weeks was slightly less than 1 for blood group A, suggesting a weak protective effect, as shown in Fig. 2.

**Table 3:** Associations of outcomes with blood group in VLBW infants.

Outcomes	Univariable model	Multivariable model 1	Multivariable model 2	Multivariable model 3
	aOR (95% CI)	aOR (95% CI)	aOR (95% CI)	aOR (95% CI)
NEC ≥ stage 2 or death				
O vs non-O	0.98 (0.84, 1.15)	0.99 (0.84, 1.17)	0.99 (0.84, 1.17)	0.99 (0.84, 1.17)
A vs non-A	1.03 (0.88, 1.22)	0.98 (0.83, 1.17)	0.98 (0.83, 1.16)	0.98 (0.83, 1.16)
B vs non-B	0.94 (0.81, 1.10)	0.97 (0.82, 1.14)	0.96 (0.82, 1.13)	0.96 (0.82, 1.13)
AB vs non-AB	1.12 (0.89, 1.42)	1.15 (0.90, 1.47)	1.16 (0.91, 1.49)	1.17 (0.92, 1.49)
NEC ≥ stage 2				
O vs non-O	1.09 (0.84, 1.40)	1.09 (0.84, 1.41)	1.10 (0.85, 1.42)	1.10 (0.85, 1.42)
A vs non-A	1.15 (0.89, 1.49)	1.10 (0.85, 1.44)	1.10 (0.85, 1.43)	1.10 (0.85, 1.43)
B vs non-B	0.88 (0.68, 1.14)	0.91 (0.70, 1.17)	0.90 (0.70, 1.17)	0.91 (0.70, 1.18)
AB vs non-AB	0.80 (0.52, 1.23)	0.82 (0.53, 1.25)	0.82 (0.53, 1.26)	0.79 (0.52, 1.22)
Death				
O vs non-O	0.93 (0.78, 1.12)	0.94 (0.77, 1.13)	0.93 (0.77, 1.13)	0.93 (0.77, 1.13)
A vs non-A	1.03 (0.86, 1.24)	0.98 (0.81, 1.19)	0.98 (0.81, 1.19)	0.98 (0.81, 1.19)
B vs non-B	0.96 (0.81, 1.15)	0.99 (0.83, 1.19)	0.99 (0.82, 1.19)	0.98 (0.82, 1.18)
AB vs non-AB	1.18 (0.91, 1.54)	1.23 (0.93, 1.61)	1.24 (0.94, 1.63)	1.27 (0.96, 1.67)

Multivariable model 1 included infant and perinatal characteristics of sex, gestational age, birth weight *z*-score, multiple gestation, antenatal corticosteroid, premature rupture of the membrane, chorioamnionitis, asphyxia.

Multivariable model 2 included model 1 plus maternal characteristics of maternal age, gestational diabetes mellitus, and gestational hypertension.

Multivariable model 3 included model 2 plus admission year.

*NEC* necrotizing enterocolitis, *aOR* adjusted odds ratio, *CI* confidential internal.

### Association of ABO blood group with NEC-related outcomes

Table 4 summarizes NEC-related outcomes, including progression to stage 3 NEC, surgical intervention, and NEC-related death. Among 288 confirmed NEC cases, the distribution by blood group was: 90 cases in group O (90/1709, 5.3%), 85 in group A (85/1550, 5.5%), 89 in group B (89/1934, 4.6%), and 24 in group AB (24/581, 4.1%). Blood group AB had the lowest rates of stage 3 NEC (33.3%) and NEC-related death (16.7%), while blood group A had the lowest rate of surgical intervention (56.5%), as shown in Table 4. No significant differences in NEC-related outcomes were observed when comparing each blood group to the other three combined, as shown in Table 5.

**Table 4:** Prevalence and odds ratio of outcomes in confirmed NEC cases by blood group.

Outcomes	NEC with blood group O	NEC with blood group A	NEC with blood group B	NEC with blood group AB
	(*n* = 90)	(*n* = 85)	(*n* = 89)	(*n* = 24)
Stage 3 NEC				
*n* (%)	40 (44.4)	33 (38.8)	33 (37.1)	8 (33.3)
aOR	Reference	0.81 (0.43, 1.51)	0.75 (0.40, 1.41)	0.62 (0.23, 1.69)
Surgical NEC				
*n* (%)	56 (62.2)	48 (56.5)	57 (64.0)	15 (62.5)
aOR	Reference	0.82 (0.44, 1.54)	1.15 (0.61, 2.18)	1.05 (0.40, 2.77)
Mortality				
*n* (%)	22 (24.4)	27 (31.8)	23 (25.8)	4 (16.7)
aOR	Reference	1.76 (0.86, 3.61)	1.22 (0.59, 2.51)	0.74 (0.22, 2.54)

Adjusted all infant characteristics, perinatal and maternal characteristics plus admission year.

*NEC* necrotizing enterocolitis, *aOR* adjusted odds ratio.

**Table 5:** Adjusted odds ratio of outcomes in confirmed NEC cases: comparison between a specific blood group and other blood groups.

Outcomes		Stage 3 NEC	Surgical NEC	Mortality
Blood group O vs non-O	O, *n*%	40 (44.4)	56 (62.2)	22 (24.4)
	non-O, *n*%	74 (37.4)	120 (60.6)	54 (27.3)
	aOR (95% CI)	1.32 (0.78, 2.23)	1.02 (0.60, 1.73)	0.73 (0.40, 1.36)
Blood group A vs non-A	A, *n*%	33 (38.8)	48 (56.5)	27 (31.8)
	non-A, *n*%	81 (39.9)	128 (63.1)	49 (24.1)
	aOR (95% CI)	0.96 (0.56, 1.65)	0.77 (0.45, 1.31)	1.66 (0.91, 3.04)
Blood group B vs non-B	B, *n*%	33 (37.1)	57 (64.0)	23 (25.8)
	non-B, *n*%	81 (40.7)	119 (59.8)	53 (26.6)
	aOR (95% CI)	0.87 (0.51, 1.49)	1.25 (0.73, 2.15)	0.98 (0.54, 1.79)
Blood group AB vs non-AB	AB, *n*%	8 (33.3)	15 (62.5)	4 (16.7)
	non-AB, *n*%	106 (40.2)	161 (61.0)	72 (27.3)
	aOR (95% CI)	0.73 (0.29, 1.86)	1.07 (0.43, 2.63)	0.58 (0.18, 1.81)

Adjusted all infant characteristics, perinatal and maternal characteristics plus admission year and non-blood group as reference.

*n*% using total case of ≥stage 2 NEC in the blood group as the denominator.

*NEC* necrotizing enterocolitis, *aOR* adjusted odds ratio, *CI* confidential internal.

In sensitivity analyses, excluding infants who died or were discharged within 7 or 14 days of life and stratifying by GA, demonstrated consistent findings for the primary composite outcome, as shown in Tables S1–3.

## Discussion

Since first proposed in 2012, blood group AB has been regarded as associated with an increased incidence and severity of NEC, as well as increased need for surgical intervention and mortality.^7,20,21^ However, this study, which included 5774 VLBW infants, did not find such associations. Compared to blood group O, there was no significant difference in the mortality and incidence of confirmed NEC (Bell’s stage ≥2) in blood group A, B, and AB. Furthermore, there were no differences in the incidence of severe NEC (Bell’s stage 3), need for surgery, or NEC-related death. Even when one blood group was compared with the other three combined, no differences were found. These results suggest that ABO blood group does not influence the incidence, severity, need for surgery, or mortality of NEC.

This discrepancy may stem from differences in cohort selection. Both the 2012^7^ and 2021^20^ studies analyzed cohorts of NEC patients and examined the distribution of ABO blood groups within these groups. The number of NEC cases in each blood group was influenced by the total population and the incidence of NEC in that group, which may have confounded the distributions of blood groups in their studies. In contrast, based on a large cohort of VLBW infants, we calculated and compared the ratio of primary outcomes in each blood group, expressed as the number of confirmed NEC cases in a blood group divided by the total number of VLBW infants in that group. This approach more accurately reflects the natural incidence of NEC in each blood group and ensures that subsequent statistical analyses are more reliable.

A 2021 study by Martynov et al.^21^ used a similar approach to ours. This multi-center study, which included 10,257 VLBW infants, concluded that blood group AB is a risk factor for surgical NEC, focal intestinal perforation, or death. Although these conditions share some clinical manifestations, focal intestinal perforation and NEC are distinct diseases, both predominantly affecting VLBW infants. Furthermore, 38.9% of the confirmed NEC cases in our study did not require surgical intervention, making Martynov et al.’s findings less comparable to ours, as our primary outcome included both confirmed NEC and mortality.

To minimize confounding factors, this study employed three multivariable models. The first model adjusted for infant and perinatal characteristics, including sex, GA, BW *z*-score, multiple gestation, antenatal corticosteroid use, premature rupture of membranes, chorioamnionitis, and asphyxia. The second model further accounted for maternal characteristics, while the third model incorporated the influence of admission year. Additionally, the impact of death or discharge against medical advice within 7 or 14 days after birth was also excluded (Tables S1–3). This stepwise approach yielded consistent results, indicating that ABO blood group does not independently influence the incidence or severity of NEC and mortality in VLBW infants.

In addition to statistical techniques, differing transfusion strategies may explain some of the discrepancies in findings. In our institution, leukocyte-depleted blood group-compatible RBCs are used for transfusions, whereas Thomson’s study^7^ utilized group O and Rh-negative RBCs for all recipients. Group O RBCs inevitably contain isoagglutinins, which may induce immune reactions, especially in recipients with blood group AB, who have antibodies against both type A and type B antigens. These immune reactions could potentially exacerbate the inflammatory cascade leading to NEC, although this hypothesis has not been experimentally validated. Transfusion with precisely matched RBCs might better eliminate the influence of isoagglutinins, providing a more “natural” epidemiological view of NEC.

ABO blood groups may influence NEC through interactions with the gut microbiota. Dysbiosis or aberrant production of microbiota metabolites has been implicated in NEC, and ABO blood group may affect the composition and growth of gut microbiota. Non-O blood groups (A, B, and AB) may secrete Fucosyltransferase 2, which serves as an adhesion receptor and energy source for Bacteroides and Faecalibacterium species in the gut.^22^ These bacteria help degrade complex plant polysaccharides into short-chain fatty acids like butyrate, which are crucial for maintaining intestinal barrier function.^23^ Bacteroides also plays a role in restoring bile acid metabolism and promoting the differentiation of regulatory T cells, thereby protecting against NEC.^24^ Moreover, Bifidobacteria, which are more abundant in the gut of non-O groups^25^, are known to protect against gut inflammation.^26,27^ Genes associated with ABO blood groups can regulate gut microbiota by influencing N-acetylglucosamine (GalNAc) levels.^28^ However, these findings are primarily based on experimental studies and have not yet been confirmed in clinical practice. Since the study population was VLBW infants, the effects of other factors such as gut immaturity, ischemia, and formula feeding may overwhelm the influence of ABO blood groups.

This study has several limitations. As a single-center study, the number of participants with blood group AB was relatively small, comprising only about 9–10% of the total population. This imbalance could introduce bias into the statistical analysis. However, the distribution of ABO blood groups in this study is quite similar to the natural distribution of the nearby provinces.^29^ Additionally, the influence of other factors, such as feeding protocols and antibiotic use, was not excluded from the analysis. Our explanations for the differences between our findings and those of previous studies are mostly hypothetical and should be further validated by future studies.

## Conclusion

There is no significant association among ABO blood groups with regard to confirmed NEC incidence, severity and mortality in VLBW infants. Different transfusion strategies may explain the discrepancies between this study and previous ones. However, these findings still require further validation through multicenter studies and experimental investigations.

## Supplementary information

Supplementary Materials

## Data Availability

The data that support the findings of this study are available from the corresponding author upon reasonable request.
