# Supplementary material for: Impact of ABO blood group on NEC incidence and mortality in VLBW infants
Source: Pediatr Res. 2025 Jun 7;99(2):686–92. doi: 10.1038/s41390-025-04181-z (PMC12956559; doi:10.1038/s41390-025-04181-z)
Supplement: Supplementary file 1 — Supplementary Materials [file 41390_2025_4181_MOESM1_ESM.pdf]

Table S1. Sensitivity analysis: Associations of outcomes with blood group in VLBW after excluding infants died or discharged within 7 days of life

**Outcomes**	**Univariable model aOR (95%CI)**	**Multivariable model 1 aOR (95%CI)**	**Multivariable model 2 aOR (95%CI)**	**Multivariable model 3 aOR (95%CI)**
**NEC ≥ stage 2 or death**				
Blood group O	Reference	Reference	Reference	Reference
Blood group A	0.93 (0.73, 1.19)	0.89 (0.70, 1.14)	0.89 (0.70, 1.14)	0.89 (0.70, 1.14)
Blood group B	0.85 (0.67, 1.07)	0.87 (0.69, 1.10)	0.87 (0.69, 1.11)	0.87 (0.69, 1.11)
Blood group AB	0.81 (0.57, 1.15)	0.83 (0.58, 1.18)	0.83 (0.58, 1.18)	0.83 (0.58, 1.18)
**NEC ≥ stage 2**				
Blood group O	Reference	Reference	Reference	Reference
Blood group A	1.05 (0.78, 1.43)	1.01 (0.75, 1.38)	1.01 (0.74, 1.38)	1.01 (0.74, 1.37)
Blood group B	0.88 (0.65, 1.19)	0.90 (0.66, 1.22)	0.90 (0.66, 1.22)	0.89 (0.66, 1.21)
Blood group AB	0.80 (0.50, 1.27)	0.82 (0.51, 1.30)	0.81 (0.51, 1.30)	0.80 (0.50, 1.27)
**Death**				
Blood group O	Reference	Reference	Reference	Reference
Blood group A	0.93 (0.68, 1.27)	0.88 (0.64, 1.21)	0.88 (0.64, 1.21)	0.88 (0.68, 1.21)
Blood group B	0.84 (0.62, 1.13)	0.87 (0.64, 1.18)	0.87 (0.64, 1.18)	0.87 (0.64, 1.19)
Blood group AB	0.77 (0.49, 1.22)	0.78 (0.49, 1.25)	0.78 (0.49, 1.25)	0.80 (0.50, 1.27)

Abbreviations: NEC, necrotizing enterocolitis; aOR, adjusted odds ratio; CI, confidential internal.

Multivariable model 1 included infant and perinatal characteristics of sex, gestational age, birth weight z-score, multiple gestation, antenatal corticosteroid, premature rupture of the membrane, chorioamnionitis, asphyxia.

Multivariable model 2 included model 1 plus maternal characteristics of maternal age, gestational diabetes mellitus, gestational hypertension.

Multivariable model 3 included model 2 plus admission year.

Table S2. Sensitivity analysis: Associations of outcomes with blood group in VLBW after excluding infants died or discharged within 14 days of life

Outcomes	Univariable model aOR (95%CI)	Multivariable model 1 aOR (95%CI)	Multivariable model 2 aOR (95%CI)	Multivariable model 3 aOR (95%CI)
**NEC ≥ stage 2 or death**				
Blood group O	Reference	Reference	Reference	Reference
Blood group A	1.05 (0.81, 1.38)	1.00 (0.76, 1.31)	1.00 (0.76, 1.31)	0.98 (0.71, 1.34)
Blood group B	0.89 (0.69, 1.16)	0.92 (0.70, 1.19)	0.92 (0.70, 1.20)	0.87 (0.64, 1.19)
Blood group AB	0.87 (0.59, 1.29)	0.88 (0.60, 1.30)	0.88 (0.60, 1.31)	0.81 (0.51, 1.29)
**NEC ≥ stage 2**				
Blood group O	Reference	Reference	Reference	Reference
Blood group A	1.03 (0.75, 1.41)	0.98 (0.72, 1.35)	0.98 (0.71, 1.34)	0.98 (0.71, 1.34)
Blood group B	0.86 (0.63, 1.18)	0.88 (0.64, 1.20)	0.90 (0.64, 1.19)	0.87 (0.64, 1.19)
Blood group AB	0.82 (0.51, 1.30)	0.83 (0.52, 1.32)	0.83 (0.52, 1.32)	0.81 (0.51, 1.29)
**Death**				
Blood group O	Reference	Reference	Reference	Reference
Blood group A	1.17 (0.80, 1.71)	1.10 (0.75, 1.61)	1.10 (0.75, 1.61)	1.10 (0.75, 1.61)
Blood group B	0.93 (0.64, 1.36)	0.97 (0.66, 1.42)	0.97 (0.66, 1.42)	0.97 (0.66, 1.43)
Blood group AB	0.87 (0.49, 1.53)	0.87 (0.49, 1.55)	0.87 (0.49, 1.55)	0.89 (0.50, 1.59)

Abbreviations: NEC, necrotizing enterocolitis; aOR, adjusted odds ratio; CI, confidential internal.

Multivariable model 1 included infant and perinatal characteristics of sex, gestational age, birth weight z-score, multiple gestation, antenatal corticosteroid, premature rupture of the membrane, chorioamnionitis, asphyxia.

Multivariable model 2 included model 1 plus maternal characteristics of maternal age, gestational diabetes mellitus, gestational hypertension.

Multivariable model 3 included model 2 plus admission year.

Table S3. Subgroup analysis: risk of outcomes in infants with gestational age less than 28 weeks after excluding infants died or discharged within 7 days or 14 days of life

	aOR(95%CI)		
	**Blood group A**	**Blood group B**	**Blood group AB**
**After excluding infants died or discharged within 7 days of life**			
NEC ≥ stage 2 or death	0.91 (0.72, 1.14)	0.90 (0.72, 1.13)	0.93 (0.67, 1.29)
NEC ≥ stage 2	1.01 (0.74, 1.37)	0.90 (0.66, 1.21)	0.78 (0.49, 1.24)
Death	0.91 (0.68, 1.21)	0.92 (0.70, 1.22)	0.98 (0.66, 1.47)
**After excluding infants died or discharged within 14 days of life**			
NEC ≥ stage 2 or death	0.97 (0.76, 1.25)	0.95 (0.75, 1.25)	0.98 (0.69, 1.38)
NEC ≥ stage 2	1.01 (0.74, 1.38)	0.88 (0.65, 1.20)	0.80 (0.50, 1.28)
Death	1.01 (0.73, 1.40)	1.01 (0.73, 1.38)	1.08 (0.69, 1.70)

Abbreviations: NEC, necrotizing enterocolitis; aOR, adjusted odds ratio; CI, confidential internal.

The reference group used for the plot is the blood group O group. Higher odds ratios indicate a great chance of outcomes. aOR(95%CI) adjusted all infant characteristics, perinatal and maternal characteristics plus admission year.
